# Supplementary material for: Sorting at embryonic boundaries requires high heterotypic interfacial tension
Source: Nat Commun. 2017 Jul 31;8:157. doi: 10.1038/s41467-017-00146-x (PMC5537356; doi:10.1038/s41467-017-00146-x)
Supplement: Supplementary file 2 — Supplementary Software 1 [file 41467_2017_146_MOESM2_ESM.zip › PottsModel/SrcPottsModel/doc/mvc/IObservable.html]

IObservable


JavaScript is disabled on your browser.


Skip navigation links


- Overview
- Package
- Class
- Use
- Tree
- Deprecated
- Index
- Help

- Prev Class
- Next Class

- Frames
- No Frames

- All Classes

- Summary:
- Nested |
- Field |
- Constr |
- Method

- Detail:
- Field |
- Constr |
- Method


mvc

## Interface IObservable

- All Known Implementing Classes:
  :   AObservable, Cell, Lattice, PottsEngine, SquareCell, StatisticsManager

  ---

    

  ```
  public interface IObservable
  ```

- - ### Method Summary

    All Methods Instance Methods Abstract Methods

    | Modifier and Type | Method and Description |
    | --- | --- |
    | `void` | `addObserver(Observer pObserver)` Add an observer to the observable object. |
    | `long` | `getNotificationSequenceNumber()` Get the notification sequence number within the source object. |
    | `java.lang.String` | `getNotificationSource()` |
    | `void` | `notifyObservers(javax.management.Notification pNotification)` Notifies the observers of this of a change in state. |

- - ### Method Detail


    - #### addObserver

      ```
      void addObserver(Observer pObserver)
      ```

      Add an observer to the observable object.

      Parameters:
      :   `pObserver` - : Observer to add


    - #### notifyObservers

      ```
      void notifyObservers(javax.management.Notification pNotification)
      ```

      Notifies the observers of this of a change in state.

      Parameters:
      :   `pNotification` - : Notification to send to observers.


    - #### getNotificationSequenceNumber

      ```
      long getNotificationSequenceNumber()
      ```

      Get the notification sequence number within the source object.

      Returns:
      :   Serial number identifying a particular instance of notification in the context of the notification source.


    - #### getNotificationSource

      ```
      java.lang.String getNotificationSource()
      ```


Skip navigation links


- Overview
- Package
- Class
- Use
- Tree
- Deprecated
- Index
- Help

- Prev Class
- Next Class

- Frames
- No Frames

- All Classes

- Summary:
- Nested |
- Field |
- Constr |
- Method

- Detail:
- Field |
- Constr |
- Method
